# Potential Hepatotoxicity of Efavirenz and Saquinavir/Ritonavir Coadministration in Healthy Volunteers

**DOI:** 10.1111/j.1753-5174.2009.00016.x

**Published:** 2009-03

**Authors:** Candice Jamois, Myriam Riek, Christophe Schmitt

**Affiliations:** F. Hoffmann-La Roche AGBasel, Switzerland

**Keywords:** Drug Interaction, Efavirenz, Human Immunodeficiency Virus, Pharmacokinetics, Ritonavir, Saquinavir

## Abstract

**Objective:**

This study was designed to investigate the pharmacokinetic effects of coadministration of saquinavir/ritonavir with efavirenz at steady state.

**Methods:**

Healthy volunteers in this open-label, two-arm, one-sequence, two-period crossover study (planned enrollment of 40 participants) were randomized to one of two treatment arms: those in Arm 1 were scheduled to receive saquinavir/ritonavir 1,000/100 mg orally twice daily for 29 days and efavirenz 600 mg orally once daily starting on day 15 and continuing through day 29; participants randomized to Arm 2 were to receive efavirenz once daily for 29 days and saquinavir/ritonavir 1,000/100 mg twice daily starting on day 15 through day 29. Assessments included vital signs, laboratory analyses, electrocardiography, and blood levels of total saquinavir, ritonavir, and efavirenz. Pharmacokinetic parameters included C_max_ (maximum observed plasma concentration), t_max_ (time to reach the maximum observed plasma concentration), 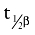 (apparent elimination half-life), and AUC_0-τ_ (area-under-the-plasma-concentration-time curve over one dosing interval).

**Results:**

Eight participants (four in each arm) were enrolled; only two (one from each treatment arm) reached day 15 of the study and received the concurrent initial doses of saquinavir/ritonavir and efavirenz. The study was terminated prematurely after these two participants experienced nonserious adverse events. The participant in Arm 1 experienced mild abdominal discomfort, diarrhea, sleep disorder, and headache and the participant in Arm 2 experienced moderate-intensity abdominal pain and mild vomiting with leukocytosis accompanied by elevated pancreatic and hepatic enzymes (aspartate aminotransferase and alanine aminotransferase values of 2-fold and 3.5-fold the upper limit of normal, respectively). Both participants recovered completely following treatment discontinuation. Only limited pharmacokinetic data were generated on these two participants.

**Conclusions:**

The early termination of this study precluded drawing any definitive conclusions regarding the pharmacokinetics at steady state of coadministered saquinavir/ritonavir and efavirenz.

## Introduction

Efavirenz (Sustiva®; Bristol-Myers Squibb) is a non-nucleoside reverse transcriptase inhibitor, antiviral agent indicated for the treatment of human immunodeficiency virus-1 (HIV-1) infection. Because of its prolonged half-life (40 to 55 hours), efavirenz can be dosed once daily [1]. Efavirenz is also a potent inducer of the cytochrome P4503A4 isoenzyme (CYP3A4) [1], the isoenzyme that metabolizes the protease inhibitor antivirals. When efavirenz is coadministered with the protease inhibitors, a profound reduction in protease inhibitor exposure results [1,2].

Saquinavir (Invirase®; Hoffmann-La Roche) is a potent HIV protease inhibitor with oral bioavailability limited by extensive first-pass metabolism mediated primarily by CYP3A4. While saquinavir is a weak CYP3A4 inhibitor itself [3], its exposure is enhanced when combined with a low (subtherapeutic) dose of ritonavir [4], a potent inhibitor of CYP3A4 [5]. Thus, increasing the plasma concentrations of saquinavir by combining it with low-dose ritonavir may compensate for the acceleration of its metabolism by efavirenz.

Few studies have evaluated the pharmacokinetics of saquinavir/ritonavir and efavirenz when coadministered. In one study of healthy participants, no significant differences in plasma concentrations of saquinavir were observed following once-daily administration of saquinavir 1,600 mg/ritonavir 200 mg when administered after a 2-week run-in period with efavirenz 600 mg or when efavirenz was added following a 2-week run-in period with saquinavir/ritonavir [6]. Similar results were reported when efavirenz 600 mg was added once daily after 10-day administration of saquinavir 400 mg/ritonavir 400 mg twice daily in healthy volunteers [7].

In an open-label efficacy study of 32 efavirenz-naive, protease inhibitor-experienced, HIV-infected patients who received efavirenz in combination with a twice-daily boosted regimen (saquinavir 1,000 mg/ritonavir 100 mg) for 6 months, there did not appear to be any clinically relevant alterations in plasma levels of saquinavir resulting from the combination (levels of other agents were not measured), although these values ranged widely [8]. Another similarly designed study evaluated the pharmacokinetics of the three agents in 42 HIV-infected patients who were switched from their current regimen to a once-daily boosted regimen (saquinavir 1,200 mg/ritonavir 100 mg) plus efavirenz due to adverse events [9]. As in the previous study, a large interpatient variability in saquinavir and efavirenz pharmacokinetics was noted, although the plasma levels of both of these agents were adequate to maintain virologic suppression [9]. Because only limited data are available to support the use of saquinavir/ritonavir with efavirenz, the present study was designed to further investigate the pharmacokinetic effects of coadministration of all three drugs at steady state.

## Methods

### Study Design

**Figure 1:**
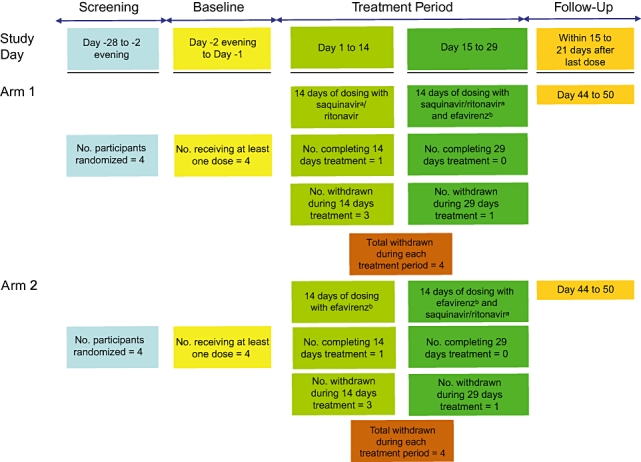
Study design and patient disposition. ^a^Saquinavir/ritonavir 1,000/100 mg doses every 12 hours (±1 hour). On Day 15, only the evening dose was administered. ^b^Efavirenz 600 mg every 24 hours at bedtime.

This open-label, randomized, two-arm, one-sequence, two-period crossover study in healthy volunteers was conducted at the Roche Clinical Pharmacology Unit in Strasbourg, France. The overall study design is shown in Figure 1. An enrollment of 40 participants (20 for each treatment arm) was planned. The study was conducted in full conformance with the principles of the Declaration of Helsinki or with the laws and regulations of the country in which the research was conducted, whichever provided participants with the greatest protection. Written informed consent was obtained from all participants before any screening procedures were performed.

### Study Participants

Healthy males or females aged 18 to 65 years with a body-mass index of 18 to 30 kg/m^2^ were eligible if on screening they had a clinically normal physical examination and laboratory tests and no prior history of: (i) clinically significant gastrointestinal, renal, hepatic, bronchopulmonary, neurological, cardiovascular, or endocrinologic disease; (ii) generalized drug reaction, including severe allergic asthma, regional or generalized urticaria, or anaphylaxis of any cause; or (iii) any major illness within 4 weeks prior to screening. Females were excluded if they were pregnant, breast-feeding, or using hormone replacement therapy. Females of childbearing potential were required to use effective nonhormonal contraception during the study and for at least 1 month after the last dose. Use of any CYP3A4 inhibitor or inducer within 4 weeks or any other medication (except aspirin or acetaminophen, allowed up to 48 hours of dosing) within 2 weeks prior to the first dose of study drug or within six times the elimination half-life (whichever was longer) was prohibited. Participants were to abstain from ingesting grapefruit/grapefruit juice within 14 days and any herbal product containing Saint John's wort or garlic within 4 weeks from dosing and from using tobacco for a minimum of 6 months prior to the start of the study. In addition, participants were excluded if they had a positive test for drugs of abuse in their urine or a positive alcohol breath test at screening or baseline; had a history of alcohol and/or drug abuse; had a positive test for HIV or hepatitis B or C at screening; had participated in a clinical study with an investigational drug within 3 months prior to dosing; and/or had donated or experienced blood loss of more than 400 mL in the 3-month period prior to dosing.

### Study Treatment

Participants randomized to Arm 1 received saquinavir/ritonavir 1,000/100 mg orally twice daily (morning and evening doses at 12-hour intervals) for 29 days (day 1 to day 29). Starting on day 15 and continuing through day 29, these participants also received efavirenz 600 mg orally once daily at bedtime (approximately 10:00 pm). Participants randomized to Arm 2 received efavirenz once daily at bedtime for 29 days and saquinavir/ritonavir 1,000/100 mg twice daily (at 12-hour intervals) starting on day 15 through day 29. To ensure that steady-state blood levels were reached for both saquinavir and ritonavir, the combination was administered for 14 days. This duration, although longer than required based on the half-lives of the individual drugs, was selected because ritonavir is not only a potent inhibitor of CYP3A but also increases the enzyme activity of CYP3A (inhibition-associated induction) [5]. Due to this autoinduction, plasma concentrations of saquinavir and ritonavir generally reach steady state 2 weeks after the start of ritonavir administration. To ensure that maximal induction potential was achieved, efavirenz also was administered for 14 days.

Randomization numbers were generated by the sponsor and allocated by the Unit's pharmacist sequentially in the order in which participants were screened and enrolled. On day 15, no morning doses of either agent were given. Saquinavir/ritonavir was administered concurrently with meals (which were standardized to fat content in the study unit) and approximately 2 hours prior to efavirenz administration.

### Clinical and Pharmacokinetic Assessments

Participants were required to stay at the study center the night prior to study initiation, on days 11, 13, 14, 15, 16, 21, 26, 29, and at follow-up. Vital signs were taken, and electrocardiography and clinical laboratory tests (hematology, biochemistry, and urinalysis) were performed at least weekly. All adverse events reported during the study were recorded and graded as to intensity. Participants presenting confirmed values of hepatic laboratory tests of more than three times the upper limit of normal (ULN), whether or not associated with clinical signs, were to be withdrawn from the study. If more than 2 subjects in one arm presented severe signs of toxicity (grade 3/4) or other safety concerns of the same kind/same organ system for which relationship to the study drugs was considered possible or probable, the discontinuation of the study arm could be decided. Discontinuation of the other study arm could also be discussed between the investigator and the clinical pharmacology scientist.

Blood levels of total saquinavir and ritonavir were determined in Arm 1 and of efavirenz in Arm 2 on days 14 and 29 at predose and 0.5, 1, 2, 3, 4, 5, 6, 8, 10, 12, 14 (16 for efavirenz), and 24 hours after the evening/bedtime dose of saquinavir/ritonavir or efavirenz. Blood levels of efavirenz in Arm 1 and of saquinavir and ritonavir in Arm 2 were also measured on day 29 at predose and at 4 hours after the evening dose. Additional blood levels were drawn before the evening/bedtime doses on days 11, 12, and 13 and on days 26, 27, and 28.

The following pharmacokinetic parameters were estimated using standard noncompartmental methods: C_max_ (maximum observed plasma concentration), t_max_ (time to reach the maximum observed plasma concentration), 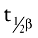 (apparent elimination half-life), and AUC_0-τ_ (area-under-the-plasma-concentration-time curve over one dosing interval). In addition, trough plasma concentrations were estimated prior to the evening dose on days 11, 12, 13, 14, 16, 27, 28, and 29. The study was designed to include additional pharmacokinetic assessments at day 29; these were not performed due to the premature termination of the study.

Plasma samples were analyzed for saquinavir and ritonavir by Pharma Bio-Research Group BV (Assen, the Netherlands) and for efavirenz by BAS Analytics (Stareton, United Kingdom) using validated, specific, high-performance liquid chromatography/mass spectroscopy/mass spectroscopy methods. The lower limit of quantification was 1 ng/mL for saquinavir and ritonavir and 200 ng/mL for efavirenz.

## Results

A total of eight participants (four in each arm), aged 26 to 45 years, of whom two were females and seven were Caucasian, were enrolled. Two participants only (one from each treatment arm) reached day 15 of the study and received the initial doses of the combination of saquinavir/ritonavir and efavirenz. The investigator together with the sponsor terminated the study prematurely after these two participants experienced nonserious adverse events, with the remaining participants in Arm 1 receiving 4, 9, and 14 days of saquinavir/ritonavir and those in Arm 2 receiving 3, 8, and 10 days of efavirenz (see Figure 1 for the disposition of the participants). One participant from Arm 2 developed a maculopapular rash on day 9 of treatment with efavirenz and withdrew from the study for this reason.

Adverse events reported by the participant from Arm 1 who received the combination of saquinavir/ritonavir and efavirenz included mild abdominal discomfort, diarrhea, sleep disorder, and headache. The study drug was discontinued and all symptoms resolved without treatment. The majority of laboratory test results remained within normal ranges, including liver function tests, pancreatic enzymes, and leukocyte count and differential. Elevated total cholesterol (5.70 mmol/L; grade 2) and triglyceride (6.27 mmol/L; grade 3) levels on day 16 normalized by day 17. Five hours after coadministration of saquinavir/ritonavir and efavirenz on the evening of day 15, the participant from Arm 2 experienced moderate-intensity abdominal pain and mild vomiting. Laboratory tests showed leukocytosis with neutrophilia accompanied by elevated pancreatic and hepatic enzymes and serum triglyceride levels. Aspartate aminotransferase levels were 77 IU/L (grade 1; 2-fold the ULN) on day 17 and alanine aminotransferase levels were 145 IU/L (grade 2; 3.5-fold the ULN) on day 18. The study drug was discontinued and the clinical symptoms were resolved without treatment on day 16. All laboratory abnormalities normalized by day 19 except for the alanine aminotransferase elevation, which declined to normal by day 29. Although the adverse events in these participants were nonserious, these elevations appeared similar in nature to the hepatotoxicity reported in a separate clinical study involving saquinavir/ritonavir and rifampin, another potent inducer of CYP3A4 [10].

All four participants in Arm 1 and three of four participants in Arm 2 reported at least one adverse event. Most of the reported events were mild in intensity, and none were reported to be severe. In addition to the adverse events described above, those considered to be probably related to study treatment were: maculopapular rash in one additional participant during treatment with efavirenz and sleep disorder in one participant during treatment with efavirenz. No participant experienced a clinically relevant QTc prolongation during the study, and no clinically significant physical abnormalities were observed.

### Pharmacokinetic Analysis

**Figure 2:**
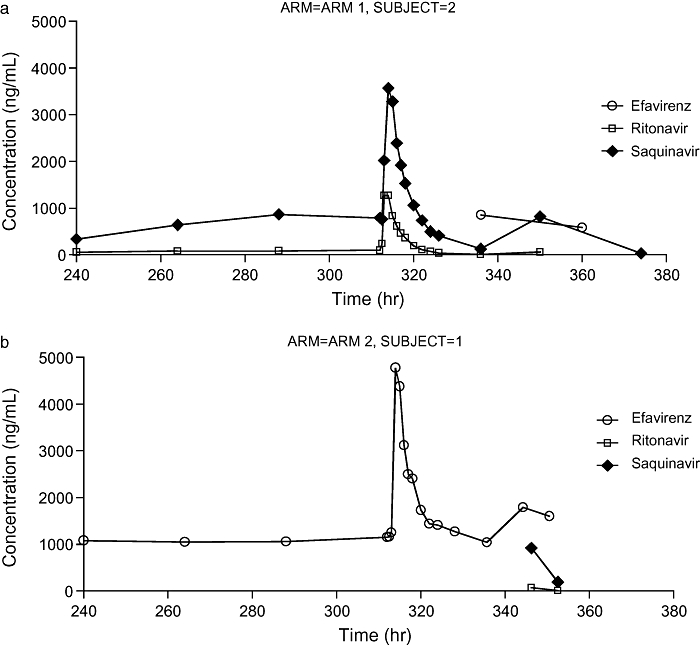
(a) Plasma concentration-time curves for participant 2, Arm 1. Saquinavir/ritonavir trough concentrations on days 11 to 13 and full pharmacokinetic (PK) profile on day 14 (312 to 336 hours). Sparse plasma concentrations from samples taken after initiation of triple-combination therapy are also shown. (b) Plasma concentration-time curves for participant 1, Arm 2. Efavirenz trough concentrations on days 11 to 13 and full PK profile on day 14 (312 to 336 hours). Sparse plasma concentrations from samples taken after initiation of triple-combination therapy are also shown.

Limited pharmacokinetic data were available from blood level data taken from three participants prior to the termination of the study. Scheduled blood samples for trough plasma level determinations were collected from two participants in Arm 1 and from one participant in Arm 2 (Table 1). The individual plasma concentration-time curves for all three agents are presented in Figure 2a, b. Complete steady-state pharmacokinetic profiles (day 14) were available for only one participant in each arm (Table 2). Pharmacokinetic profiles were not obtainable for the two participants who received all three agents; only sparse blood samples were taken after their coadministration as these two participants withdrew from the study on day 15. However, the plasma concentrations of saquinavir, ritonavir, and efavirenz determined in these samples were similar to concentrations observed during the administration of saquinavir/ritonavir and efavirenz alone in these participants (Figure 2a, b).

**Table 1:** Individual trough plasma concentrations, ng/mL

	Day 11	Day 12	Day 13
Saquinavir			
Arm 1, participant 2	337	640	867
Arm 1, participant 4	602	700	1,220
Ritonavir			
Arm 1, participant 2	57.8	83.8	86.2
Arm 1, participant 4	137	163	251
Efavirenz			
Arm 2, participant 1	1,080	1,050	1,060

**Table 2:** Pharmacokinetic parameters at steady state in the two participants experiencing toxicity

Participant Treatment Arm	Drug	C_max_ (ng/mL)	t_max_ (h)	AUCo-τ (ng*h/mL)	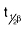 (h)
Arm 2, participant 1	Efavirenz	4,780	2.00	42,523	26.5
Arm 1, participant 2	Ritonavir	1,270	1.00	5,512	3.53
Arm 1, participant 2	Saquinavir	3,570	2.00	19,640	5.98

τ, Twenty-four hours for efavirenz and 12 hours for saquinavir/ritonavir.

AUCo-τ = area-under-the-plasma-concentration-time curve over one dosing interval; C_max_ = maximum observed plasma concentration; t_max_ = time to reach the maximum observed plasma concentration; 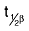 = apparent elimination half-life.

## Discussion

The hepatic and gastrointestinal adverse events identified in two participants in this study were unanticipated based on previously reported studies in which saquinavir/ritonavir and efavirenz were coadministered in healthy participants or in HIV-infected patients, although safety data are limited [6–9]. Commonly reported adverse events have included dizziness [6,7] and headache [6], and no clinically relevant alterations in laboratory parameters were reported in the majority of studies [6–8] although one HIV-infected patient with chronic hepatitis B and C had liver enzyme elevations due to hepatitis reactivation [9]. In a study involving 11 HIV-infected patients on a regimen of saquinavir 1,000 mg/ritonavir 200 mg twice daily in combination with efavirenz 600 mg once daily, one patient developed grade 4 hepatotoxicity; therapy was successfully resumed with lower doses of ritonavir (100 mg) and efavirenz (400 mg) [11]. A review of the Roche Drug Safety database for adverse events associated with the use of saquinavir/ritonavir with efavirenz compared to saquinavir/ritonavir without efavirenz revealed too few cases on the triple combination, precluding a meaningful analysis.

Although this study permitted only limited pharmacokinetic analysis, the single participant receiving 2 weeks of treatment with efavirenz 600 mg once daily had pharmacokinetic parameters within previously reported ranges for HIV-infected individuals receiving efavirenz alone [12] or in combination with saquinavir [9]. Similarly, the pharmacokinetic parameters in the participant receiving saquinavir/ritonavir for 2 weeks were in agreement with those previously reported in healthy volunteers [13] or in HIV-infected patients receiving concurrent efavirenz [9]. However, due to the early termination of this study, these data are too limited to allow definitive conclusions regarding the pharmacokinetics at steady state of coadministered saquinavir/ritonavir and efavirenz.
